# Supplementary material for: Allometric shell growth in infaunal burrowing bivalves: examples of the archiheterodonts Claibornicardia paleopatagonica (Ihering, 1903) and Crassatella kokeni Ihering, 1899
Source: PeerJ. 2018 Jun 19;6:e5051. doi: 10.7717/peerj.5051 (PMC6014312; doi:10.7717/peerj.5051)
Supplement: Data S1 — Data from material used in this study, including both species, Claibornicardia paleopatagonica (Ihering, 1903) and Crassatella kokeni (Ihering, 1899). Collection abbreviations mentioned in the main manuscript. [file peerj-06-5051-s001.docx]

**Supplemental Data S1**

*Sample*

Data from material used in this study, including both species, *Claibornicardia paleopatagonica* (Ihering, 1903) and *Crassatella kokeni* Ihering, 1899). Collection abbreviatures mentioned in the main manuscript.

| **Catalog numbers** | **Amount of specimens** | **Geography** | **Stratigraphy** |
| --- | --- | --- | --- |
| MACN-Pi 5197 | 15 specimens (including *Venericardia camachoi* Vigilante, 1977) | Puesto Ramírez, Chubut Province | Salamanca Fm. (early Danian) |
| MACN-Pi 325 | 8 specimens | Mouth of Santa Cruz River | Monte León Fm. (early Miocene) |
| MACN-Pi 326 | 3 specimens | Cabo Tres Puntas | San Julián Fm. (late Oligocene) |
| MACN-Pi 327 | 2 specimens | Punta Casamayor | San Julián Fm. (late Oligocene) |
| MACN-Pi 331 | 1 specimen | Mouth of Santa Cruz River | Monte León Fm. (early Miocene) |
| MACN-Pi 332 | 2 specimens (holotype of *Crassatellites patagonicus* Ihering, 1907) | Camarones | Camarones Fm. (middle Miocene) |
| MACN-Pi 3576 | 1 specimen | Mouth of Santa Cruz River | Monte León Fm. (early Miocene) |
| MACN-Pi 3600 | 1 specimen | Mouth of Santa Cruz River | Monte León Fm. (early Miocene) |
| MACN-Pi 3907 | 1 specimen | Cabo Tres Puntas | San Julián Fm. (late Oligocene) |
| MACN-Pi 4775 | 1 specimen | Monte Entrada | Monte León Fm. (early Miocene) |
| MACN-Pi 5374 | 1 specimen | Cañadon de los Artilleros | Monte León Fm. (early Miocene) |
| MACN-Pi 5375 | 1 specimen | Estancia Los Manantiales | Monte León Fm. (early Miocene) |
| MACN-Pi 5376 | 11 specimens | Cañadón de los Misioneros | Monte León Fm. (early Miocene) |
| CIRGEO-PI 1501 | 2 specimens | Lote 39 | Puerto Madryn Fm. (late Miocene) |
| CIRGEO-PI 1502 | 2 specimens | Lote 39 | Puerto Madryn Fm. (late Miocene) |
| CPBA 9404 | 1 specimen | Estancia Los Manantiales | Monte León Fm. (early Miocene) |
