# Supplementary material for: Allometric shell growth in infaunal burrowing bivalves: examples of the archiheterodonts Claibornicardia paleopatagonica (Ihering, 1903) and Crassatella kokeni Ihering, 1899
Source: PeerJ. 2018 Jun 19;6:e5051. doi: 10.7717/peerj.5051 (PMC6014312; doi:10.7717/peerj.5051)
Supplement: Data S4 — Includes results of PCA and URA analyses. [file peerj-06-5051-s004.docx]

**Supplemental Data S4**

*Claibornicardia paleopatagonica* (Ihering, 1903)

*Results from PCA:*

| PC | Eigenvalue | % variance | Acumulative |
| --- | --- | --- | --- |
| 1 | 8,74E+10 | 46,55 | 46,55 |
| 2 | 3,79E+10 | 20,164 | 66,714 |
| 3 | 1,37E+10 | 7,298 | 74,012 |
| 4 | 1,04E+10 | 5,5133 | 79,5253 |
| 5 | 8,14E+09 | 4,3337 | 83,859 |
| 6 | 6,03E+09 | 3,2117 | 87,0707 |
| 7 | 3,90E+09 | 2,0755 | 89,1462 |
| 8 | 3,81E+09 | 2,0289 | 91,1751 |
| 9 | 3,00E+09 | 1,5958 | 92,7709 |
| 10 | 2,69E+09 | 1,4316 | 94,2025 |
| 11 | 2,32E+09 | 1,2376 | 95,4401 |
| 12 | 1,19E+09 | 0,63276 | 96,07286 |
| 13 | 1,14E+09 | 0,60657 | 96,67943 |
| 14 | 9,37E+08 | 0,49892 | 97,17835 |
| 15 | 8,13E+08 | 0,43292 | 97,61127 |
| 16 | 6,88E+08 | 0,36648 | 97,97775 |
| 17 | 6,73E+08 | 0,35824 | 98,33599 |
| 18 | 5,66E+08 | 0,30141 | 98,6374 |
| 19 | 4,47E+08 | 0,23787 | 98,87527 |
| 20 | 3,74E+08 | 0,19923 | 99,0745 |
| 21 | 2,95E+08 | 0,15693 | 99,23143 |
| 22 | 2,63E+08 | 0,14013 | 99,37156 |
| 23 | 2,35E+08 | 0,12531 | 99,49687 |
| 24 | 1,68E+08 | 0,089223 | 99,586093 |
| 25 | 1,51E+08 | 0,080188 | 99,666281 |
| 26 | 1,35E+08 | 0,072006 | 99,738287 |
| 27 | 1,18E+08 | 0,062565 | 99,800852 |
| 28 | 7,92E+07 | 0,042193 | 99,843045 |
| 29 | 7,67E+07 | 0,040829 | 99,883874 |
| 30 | 5,84E+07 | 0,031097 | 99,914971 |
| 31 | 5,25E+07 | 0,027974 | 99,942945 |
| 32 | 3,25E+07 | 0,017303 | 99,960248 |
| 33 | 2,40E+07 | 0,012774 | 99,973022 |
| 34 | 1,82E+07 | 0,0097159 | 99,9827379 |
| 35 | 1,39E+07 | 0,0074209 | 99,9901588 |
| 36 | 1,04E+07 | 0,0055254 | 99,9956842 |
| 37 | 8,60E+06 | 0,0045769 | 100,0002611 |

*Comple results from URA:*

| Variable | Estimate | Std. Error | t value | Pr |
| --- | --- | --- | --- | --- |
| Intercept | -387001,83 | 42230,97 | -9,164 | 5.24E-13 |
| Area | 763,09 | 71,01 | 10,758 | 1,25E-15 |

Residual Standard Error: 174200 on 60 degrees of freedom

Multiple R-squared: 0,6586

Adjusted R-squared: 0,6529

F-statistic: 115,7 on 1 and 60 DF

p-value: 1,248E-15
