# Supplementary material for: Allometric shell growth in infaunal burrowing bivalves: examples of the archiheterodonts Claibornicardia paleopatagonica (Ihering, 1903) and Crassatella kokeni Ihering, 1899
Source: PeerJ. 2018 Jun 19;6:e5051. doi: 10.7717/peerj.5051 (PMC6014312; doi:10.7717/peerj.5051)
Supplement: Data S5 — Includes results of PCA and URA analyses. [file peerj-06-5051-s005.docx]

**Supplemental Data S5**

*Crassatella kokeni* Ihering, 1899

*Results from PCA:*

| PC | Eigenvalue | % variance | Acumulative |
| --- | --- | --- | --- |
| 1 | 2,76E+11 | 65,349 | 65,349 |
| 2 | 8,47E+10 | 20,098 | 85,447 |
| 3 | 2,07E+10 | 4,911 | 90,358 |
| 4 | 1,17E+10 | 2,7687 | 93,1267 |
| 5 | 6,89E+09 | 1,6331 | 94,7598 |
| 6 | 5,96E+09 | 1,4145 | 96,1743 |
| 7 | 3,38E+09 | 0,80164 | 96,97594 |
| 8 | 2,72E+09 | 0,64515 | 97,62109 |
| 9 | 1,90E+09 | 0,45102 | 98,07211 |
| 10 | 1,67E+09 | 0,39497 | 98,46708 |
| 11 | 1,51E+09 | 0,35857 | 98,82565 |
| 12 | 1,29E+09 | 0,30704 | 99,13269 |
| 13 | 7,55E+08 | 0,17913 | 99,31182 |
| 14 | 6,59E+08 | 0,15628 | 99,4681 |
| 15 | 4,67E+08 | 0,11067 | 99,57877 |
| 16 | 3,41E+08 | 0,080793 | 99,65956 |
| 17 | 3,15E+08 | 0,074618 | 99,73418 |
| 18 | 2,84E+08 | 0,067259 | 99,80144 |
| 19 | 2,37E+08 | 0,056272 | 99,85771 |
| 20 | 1,70E+08 | 0,040316 | 99,89803 |
| 21 | 1,13E+08 | 0,026866 | 99,92489 |
| 22 | 1,10E+08 | 0,025986 | 99,95088 |
| 23 | 9,39E+07 | 0,022273 | 99,97315 |
| 24 | 7,55E+07 | 0,017911 | 99,99106 |
| 25 | 3,97E+07 | 0,009414 | 100,0005 |

*Complete results from URA:*

| Variable | Estimate | Std. Error | t value | Pr |
| --- | --- | --- | --- | --- |
| Intercept | -208871,5 | 4472,5 | -4,670 | 1,36E-05 |
| Area | 879,3 | 147,2 | 5,971 | 8,15E-08 |

Residual Standard Error: 239700 on 72 degrees of freedom

Multiple R-squared: 0,3312

Adjusted R-squared: 0,3219

F-statistic: 35,66 on 1 and 72 DF

p-value: 8,149E-08
